# Georeferenced sighting and specimen occurrence data of the extinct Carolina Parakeet (*Conuropsis carolinensis*) from 1564 - 1944

**DOI:** 10.3897/BDJ.6.e25280

**Published:** 2018-06-19

**Authors:** Kevin R. Burgio, Colin J. Carlson, Alexander L. Bond

**Affiliations:** 1 University of Connecticut, Storrs, United States of America; 2 University of Maryland, Annapolis, MD, United States of America; 3 Georgetown University, Washington, D.C., United States of America; 4 Bird Group, The Natural History Museum, Tring, United Kingdom

**Keywords:** Carolina parakeet, *Conuropsis carolinensis*, distribution, extinction, Psittaciformes, North America

## Abstract

**Background:**

Despite much present-day attention on recently extinct North American birds species, little contemporary research has focused on the Carolina parakeet (*Conuropsis carolinesis*). While the last captive Carolina parakeet died 100 years ago this year, the Carolina parakeet was officially declared extinct in 1920, but they likely persisted in small, isolated populations until at least the 1930s, and perhaps longer. How this once wide-ranging and plentiful species went extinct remains a mystery. Here, we present a georeferenced dataset of Carolina parakeet sightings spanning nearly 400 years by combining both written observations and specimen data.

**New information:**

Because we include both observations and specimen data, the Carolina parakeet occurrence dataset presented here is the most comprehensive and rigorous datsetset on this species available. The dataset includes 861 sightings from 1564 to 1944. Each datapoint includes geographic coordinates, a measurement of uncertainty, detailed information about each sighting, and an assessment of the sighting's validity. Given that this species is so poorly understood, we make these data freely available to facilitate more research on this colorful and charismatic species.

## Introduction

The Carolina parakeet (*Conuropsis carolinesis*) was the only endemic North American parrot species north of the Rio Grande. The species likely went extinct in the first half of the 20th century (Snyder and Russell 2002, Elphick et al. 2010), though the proximate and ultimate causes of their extinction remain unknown. Despite being a charismatic and colorful bird, the Carolina parakeet has not received as much attention as other recently extinct North American birds, such as the passenger pigeon (*Ectopistes migratorius*, e.g. Stanton 2014), the ivory-billed woodpecker (*Campephilus principalis*, e.g. *Gotelli et al. 2011*), or Bachman's warbler (*Vermivora bachmanii*, e.g. Carlson et al. 2018). Aside from the efforts of two individual researchers, Daniel McKinley (e.g. McKinley 1965, McKinley and Hardy 1985) and Noel Snyder (Snyder and Russell 2002, Snyder 2004), there has been relatively little research on the Carolina parakeet since the last captive individual died in the Cincinnati Zoo in 1918. However, with the progression of increasingly sophisticated analytical approaches, such as genetic sequencing and species distribution modeling, researchers are starting to provide new insights into Carolina parakeets' evolutionary relationships within the parrot phylogeny (Kirchman et al. 2012) and the range size and migratory behavior (Burgio et al. 2017). To facilitate more research on this understudied, extinct species, we present the most detailed dataset yet compiled of georeferenced observations and specimens of the Carolina parakeet, ranging from 1564 to 1944.

It was only through Daniel McKinley's efforts were we able to compile this dataset. A vast majority of observations were detailed, with primary sources, in McKinley's numerous publications from 1959 to 1985 (e.g. McKinley 1965, McKinley and Hardy 1985). Within our dataset, we include observations gleaned from a wide variety of sources, including hunters' logs, the correspondence of well-known historical figures including Thomas Jefferson (Jefferson 1894) and the explorers Lewis and Clark (Lewis and Clark 1904), and even letters to the editor of local newspapers. However, some sightings may be fraudulent, as collectors from the early 20th century would often pay high prices for the eggs of a nearly extinct bird. As an example, included in the dataset is the date and location of one such potentially fradulant clutch of eggs, collected by a prolific egg collector and dealer, Charles Doe, who was notorious for passing off eggs of one species for those of other, less rare species (Snyder 2004, Snyder and Russell 2002). Fraud surrounding the Carolina parakeet was not uncommon, as evidenced by a video of living parrots painted to look like Carolina parakeets mixed in with taxidermied specimens that began circulating in the 1950s (Snyder et al. 2010). While we include these "sightings" in our dataset, they are flagged as likely invalid.

Adding to the lore surrounding Carolina parakeet observations, as late as the 1940s, ornithologist Oscar Bayard claimed that he knew of a remnant population of Carolina parakeets in rural Florida, but he refused to tell anyone where the birds were for fear that people would trap or kill the birds (Snyder 2004). In an effort to try to find information of this long lost population of Carolina parakeets, Snyder even visited his family to see if any journals were left in his affects after he died, yet he found nothing (Snyder 2004).

While data about Bayard's "lost population" has disappeared, some data may still be buried in obscure texts, waiting to be found. For instance, McKinley, who spent over 25 years writing about Carolina parakeets focusing primarily on the sightings recorded in each state, referenced two manuscripts in his writing that were never published, including a detailed account of sightings of Louisiana. We contacted his former university department to see if any of his writing or materials remained after his passing but his former collleagues could not find anything. We included as many observations as we could find from Louisiana, but it remains one of the most poorly represented states in our dataset. If other researchers find new citations or localities for the Carolina parakeet not found here, we encourage them to share them with us so we can update this dataset.

These data can be used in many avenues in research for this poorly understood species. In conjuction with modeling approaches, they may be able to help shed new light on the likely cause of the species' extinction, which remains a mystery. Accurate and precise georeferenced specimen data can be used in combination with genetic and stable isotope sampling, for example, to learn more about the relationship between the two subspecies and their dietary composition or foraging behaviour. Of the known extinct parrots, the Carolina parakeet had the largest range, by far (Olah et al. 2016). Learning how and why they went extinct may yield lessons that can inform modern conservation efforts for other parrot species, which is of pressing need, since parrots are the most-threatened major order of birds (Olah et al. 2016) with many obstacles related to conservation prioritization, especially when considering the future effects of climate change.

## Sampling methods

### Sampling description

We collected and georeferenced locality data from Carolina parakeet specimens found in natural history collections around the world (n = 460) using online data repositories, and by contacting museums directly. We also compiled observations of Carolina parakeets from scientific literature, travel diaries, and other resources from 1564 to 1944 (n = 401). Specimens and observations were georeferenced using guidelines established by Chapman and Wieczorek (2006), and the georeferencing software GEOLocate (Rios and Bart 2010). Given that place names and geographical extents of towns and water bodies have changed a great deal in the past few hundred years in North America, we paid special attention to historically-relevant maps and field journals of specimen collectors when selecting coordinates and measuring uncertainty. We include all metadata, links to historic maps used, and relevant citations associated with each sighting.

Rather than using most existing coordinates associated with museum specimens found in online data repositories (e.g., VertNet), we re-estimated the geographical coordinates of almost every specimen based on collection locality names in order to ensure consistency throughout the dataset and remove the possibility of previous errors. Perhaps most importantly, re-estimating the coordinates allowed us to measure the geographic uncertainty for each sighting. However, we did not re-estimate coordinates and uncertainty for specimens that were already measured using the same guidelines we used, though these comprise a very small percentage of the dataset (n = 4). While more Carolina parakeet specimens exist in natural history museums and collections, many are either missing locality information or the locality information was not sufficient to georeference with confidence. Because the Carolina parakeet was unique among the North American avifauna, being the only parrot species (in addition to their bold yellow and red head, and raucous calls), we were confident that misidentification was unlikely in the published accounts of the observations.

In the dataset, we include reported sightings that experts consider dubious or questionable. We also include sightings of what are likely vagrant birds (from Colorado, Maryland, Michigan, New Jersey, New York, North Dakota, Pennsylvania, and South Dakota; see Burgio et al. 2017 for more information). Additionally, we kept all georeferenced points in the dataset, regardless of the size of the estimated uncertainty associated with the coordinates. As such, these data should not be used uncritically.

## Geographic coverage

### Description

**Figure 1.**
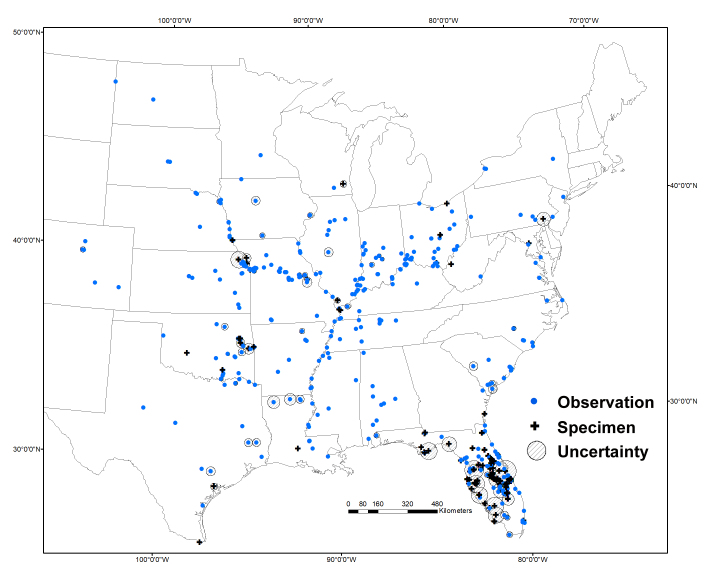
Historic sightings of the Carolina parakeet (1564-1944). Blue dots represent the location of observations recorded in the literature and black crosses represent the location specimens were collected. Circles with diagnoal lines show estimates of uncertainty associated with each point.

The Carolina parakeet has two named subspecies differentiated by geographic range: *C. c. carolinensis* which occurred along coastal southeastern United States, and the more westerly *C. c. ludovicianus* (colloqiually known as the Louisiana parakeet), which had a range from central Texas to Nebraska, west to Ohio, and south to Louisiana, with little overlap with the eastern subspecies (Burgio et al. 2017). The two subspecies were largely divided by the Appalachian mountains, only coming into contact in the area around Mississippi and Alabama (Ridgway 1916, Swenk 1934). This division is consistent with the labels of all 261 existing museum specimens that have been assigned to subspecies. Our dataset has controversial and vagrant sightings well outside of their range, with the most distant observations in Colorado, on the border of Montana and North Dakota, to Wisconsin, and northern New York (Fig. 1).

### Coordinates

25.211111 and 47.99917 Latitude; -105.222778 and -74.20528 Longitude.

## Taxonomic coverage

### Taxa included

**Table:** 

Rank	Scientific Name	
kingdom	Animalia	
phylum	Chordata	
class	Aves	Birds
order	Psittaciformes	Parrots
family	Psittacidae	African & New World parrots
tribe	Arini	Macaws and parakeets
species	*Conuropsis carolinensis*	Carolina parakeet

## Temporal coverage

**Data range:** 1564-1-01 – 1944-12-31.

### Notes

Some dates lack daily and/or monthly precision. Some sightings have no date data.

## Usage rights

### Use license

Creative Commons Public Domain Waiver (CC-Zero)

### IP rights notes

All data CC-Zero except when noted in the dataset.

## Data resources

### Data package title

Georeferenced sighting and specimen occurrence data of the extinct Carolina Parakeet (Conuropsis carolinensis) from 1564-1944

### Resource link

https://figshare.com/s/2418adbd9d87cef842d3

### Alternative identifiers

DOI: https://doi.org/10.6084/m9.figshare.5967028

### Number of data sets

1

### Data set 1.

#### Data set name

Burgio_et_al_2018_Carolina_Parakeet_Occurence_Data

#### Data format

Darwin Core Archive

#### Number of columns

30

#### Download URL

https://figshare.com/s/2418adbd9d87cef842d3

#### Description

To facilitate broad use of these data, we formatted the dataset in accordance with Darwin Core standards (Wieczorek et al. 2012). See http://rs.tdwg.org/dwc/terms/. We used Darwin Core standard column descriptions below with additional notes specific to these data. We also provide citations for all references in the "associatedReferences" column, in the dataset in a separate .txt document. We also provide an index of the abbreviations found in the "institutionCode" field, with the full name of the collection / natural history museum in which the specimen is location as a .txt document.

**Data set 1.** 

Column label	Column description
occurrenceID	An identifier for the Occurrence (as opposed to a particular digital record of the occurrence). In the absence of a persistent global unique identifier, construct one from a combination of identifiers in the record that will most closely make the occurrenceID globally unique. In this dataset, specimens use the ID number from its holding facilty; observations have their own unique observation ID.
eventDate	The date-time or interval during which an Event occurred. We present eventDate in ISO 8601 format. In some cases, day, month, or year data is missing.
year	The four-digit year in which the Event occurred, according to the Common Era Calendar.
locality	The specific description of the place. Less specific geographic information can be provided in other geographic terms (stateProvince, county). This term may contain information modified from the original to correct perceived errors or standardize the description.
stateProvince	The name of the next smaller administrative region than country (state, province, canton, department, region, etc.) in which the locality occurs.
county	The full, unabbreviated name of the next smaller administrative region than stateProvince (county) in which the locality occurs.
decimalLatitude	The geographic latitude (in decimal degrees, using the spatial reference system given in geodeticDatum) of the geographic center of a Location. Note the coordinates are very pricise, as they describe the very center of a polygon that encompasses all uncertainty associated with the locality (see coordinateUncertaintyInMeters).
decimalLongitude	The geographic longitude (in decimal degrees, using the spatial reference system given in geodeticDatum) of the geographic center of a Location. Note the coordinates are very pricise, as they describe the very center of a polygon that encompasses all uncertainty associated with the locality (see coordinateUncertaintyInMeters).
coordinateUncertaintyInMeters	The horizontal distance (in meters) from the given decimalLatitude and decimalLongitude describing the smallest circle containing the whole of the Location.
geodeticDatum	The ellipsoid, geodetic datum, or spatial reference system (SRS) upon which the geographic coordinates given in decimalLatitude and decimalLongitude as based. Data presented here in WGS84.
coordinatePrecision	A decimal representation of the precision of the coordinates given in the decimalLatitude and decimalLongitude.
georeferencedBy	A list (concatenated and separated) of names of people, groups, or organizations who determined the georeference (spatial representation) for the Location. In this case, K.R. Burgio, georeferenced all sightings but 4.
georeferencedDate	The date on which the Location was georeferenced in ISO 8601 format.
georeferenceSources	A list (concatenated and separated) of maps, gazetteers, or other resources used to georeference the Location, described specifically enough to allow anyone in the future to use the same resources.
georeferenceProtocol	A description or reference to the methods used to determine the spatial footprint, coordinates, and uncertainties. We used the GBIF best practices guidelines established by Chapman & Wieczorek (2006).
georeferenceRemarks	Notes or comments about the spatial description determination, explaining assumptions made in addition or opposition to the those formalized in the method referred to in georeferenceProtocol.
institutionCode	The name (or acronym) in use by the institution having custody of the object(s) or information referred to in the record. We include a list of abbreviation codes with the full name of the instutition in a separate .txt file with the dataset.
basisOfRecord	The specific nature of the data record.
recordedBy	A list (concatenated and separated) of names of people, groups, or organizations responsible for recording the original Occurrence. The primary collector or observer, especially one who applies a personal identifier (recordNumber), should be listed first.
associatedReferences	A list (concatenated and separated) of identifiers (publication, bibliographic reference, global unique identifier, URI) of literature associated with the Occurrence.
infraspecificEpithet	The name of the lowest or terminal infraspecific epithet. Here, we we relate subspecies identification as recorded on the specimen tag, if known.
eventRemarks	Comments or notes about the Event.
dynamicProperties	Assessment of quality of the sighting, based on expert opinion (see associated referenced for discussion of each sighting). The categories are: 1) "validity=Specimen" which indicates that a specimen exists, or did exist at one time. 2) "validity=Confirmed observation” which indicates that the observation is largely considered valid by either McKinley and/or Snyder. 3) “validity=Unconfirmed” is an observation that for one reason or another is considered controversial. 4) “vality=Likely invalid” is an observation or specimen that may be a result of fraud or mislabeling, pending independent verification.
collectionCode	The name, acronym, coden, or initialism identifying the collection or data set from which the record was derived.
catalogNumber	An identifier (preferably unique) for the record within the data set or collection.
license	A legal document giving official permission to do something with the resource.
rightsHolder	A person or organization owning or managing rights over the resource.
accessRights	Information about who can access the resource or an indication of its security status. Access Rights may include information regarding access or restrictions based on privacy, security, or other policies.
bibliographicCitation	A bibliographic reference for the resource as a statement indicating how this record should be cited (attributed) when used. Recommended practice is to include sufficient bibliographic detail to identify the resource as unambiguously as possible.
references	A related resource that is referenced, cited, or otherwise pointed to by the described resource.
